# Health literacy profiling in persons with psoriasis – A cluster analysis

**DOI:** 10.1002/ski2.17

**Published:** 2021-02-18

**Authors:** M.H. Larsen, Å. Hermansen, C.R. Borge, Y. Staalesen Strumse, M.H. Andersen, A.K. Wahl

**Affiliations:** ^1^ Lovisenberg Diaconal University College Oslo Norway; ^2^ Department of Interdisciplinary Health Sciences Institute of Health and Society University of Oslo Oslo Norway; ^3^ Faculty of Social Sciences Oslo Metropolitan University Oslo Norway; ^4^ Department of Patient Safety and Research Lovisenberg Diaconal Hospital Oslo Norway; ^5^ Section for Climate Therapy Oslo University Hospital Oslo Norway

## Abstract

**Objective:**

To explore health literacy (HL) profiles within a cohort of people with psoriasis. A cluster approach identifies groups of individuals that have similar HL profiles. The method unmasks sub‐groups with particular HL strengths, or subgroups with limitations, which require tailored healthcare services to improve.

**Methods:**

A cross‐sectional sample of 792 patients from the Norwegian Climate Helio Therapy Programme in Gran Canaria participated. The HL questionnaire assessed nine HL dimensions. Using Ward's Hierarchical Clustering Method (Stata version 16), we looked for subgroups of patients across the dimensions. We also explored whether these clusters had specific demographic features and associations to outcomes such as psoriasis knowledge, quality of life and self‐management capacity.

**Result:**

The analysis revealed four unique clusters identifying clinically meaningful subgroups. Two groups stood out as especially interesting. One cluster representing 26.6% of the sample presented severe HL limitations associated with lower psoriasis knowledge, quality of life, self‐management and self‐efficacy. HL domains connected to cooperation with healthcare professionals showed deficient scores. The other cluster included a smaller percentage (7.7%) with high HL compared to the total sample. This cluster was associated with higher self‐management, quality of life and better self‐efficacy.

**Conclusion:**

The cluster analysis revealed substantial differences in HL profiles within the sample. These results support the importance of a holistic understanding of the HL needs and the vulnerabilities within a psoriasis cohort. Implementing one size fits all approaches, may not be sufficient in psoriasis context to target HL.

## Graphical abstract

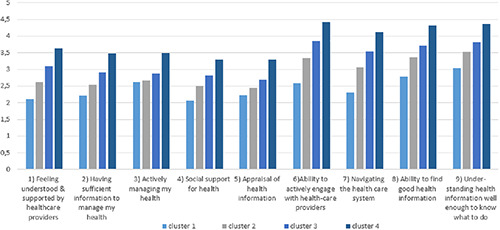

To explore whether patients with psoriasis have different strengths and limitations in their health literacy (HL) needs, we conducted cluster analyses using the Health Literacy Questionnaire. The analyses created four clear subgroups with similar HL strengths and limitations in a population of 792 Norwegians with psoriasis. The results showed large differences in HL between the clusters with associations to various demographic features, self‐management, quality of life, and psoriasis knowledge.

1

**What is already known about this topic?**

- HL is an essential factor for ensuring effective self‐management of chronic conditions such as psoriasis.
- People with psoriasis have lower HL scores compared to other chronic conditions.
- Holistic care of people with psoriasis requires knowledge about HL, self‐management support, and management of comorbidities and associated risk factors.

**What does this study add?**

- There are considerable differences in HL profiles within a psoriasis sample; having a low HL profile is associated with lower psoriasis knowledge, quality of life, self‐efficacy and self‐management.
- People in low scoring clusters are not active information seekers, have low social support and have limited faith in building relationships with healthcare providers.
- Our findings suggest that knowing HL profiles can guide the development of tailored HL interventions, securing high utility and uptake in the psoriasis context.

## 2 INTRODUCTION

Psoriasis is a chronic inflammatory skin disease, and in later years, knowledge about pathogenesis and effective pharmacological treatment options has advanced significantly. Still, several critical knowledge gaps remain, and many patients lack an efficient treatment regime^1^ ^,^ ^2^ One such knowledge gap is related to profiling the ability of patients to make use of health information related to psoriasis, named health literacy (HL).

HL refers to a person's ability to engage effectively with health information and services,^3^ and is a multidimensional concept covering functional, social and critical dimensions.^4^ Poor HL creates barriers to understand one's health, illness and treatment fully. In the HL field, most of the research has been focusing on reading comprehension and numeracy skills, better known as ‘functional’ HL.^5^ However, measuring only functional HL overlooks the complexity of cultural and personal values, the importance of context, and the social resources and individual motivation that influence peoples’ ability to understand and act upon information associated with their health.^6^

Previous findings from this sample^7^ showed that the participants with psoriasis generally scored low on most of the HL domains, also compared to people with other chronic conditions.^8^, ^9^, ^10^ However, these results provided only information of the whole sample, and the linear regression models gave no indications to whether there were significant HL differences within the psoriasis cohort. To be able to examine the Health Literacy Questionnaire (HLQ) data^10^ and reveal possible subgroups of participants, to use latent profiles or cluster analysis (CA) is recommended.^11^ Conceptually, CA aims to identify cluster solutions that are relatively homogeneous within each group, leading to clusters that show high intra‐class similarity, while maximizing heterogeneity between the groups, leading to low inter‐class similarity across the clusters.^12^

Hence, this study aimed to provide detailed profiles of HL strengths and weaknesses in the psoriasis cohort. The following research question is asked:

What types of HL profiles can be identified by investigating HLQ clusters and their characteristics in a sample of patients with psoriasis?

## 3 METHODS

### 3.1 Patients and methods

A total of 792 participants >18 years (65% response rate) provided sufficient data to be included in the CA. They had previously (once or several times from 2011 to 2017) participated in the Norwegian Climate Heliotherapy (CHT) programme in Gran Canaria. They were by postal mail requested to partake, and a reminder letter was sent after 6 weeks. Data collection took place from March to August 2017.

### 3.2 The Climate Therapy Programme

Climate therapy/heliotherapy (CHT) comprises sunlight and saltwater treatment to relieve symptoms and is one of the therapeutic options available to Norwegian patients with moderate to severe psoriasis. CHT is provided in the Canary Islands (located in the Atlantic Ocean at 28°N, 16°W) and includes 3 weeks of individualized sun exposure in increasing doses as the primary treatment. Additionally, the programme emphasizes daily physical training, tailored education, group discussions, individual consultations, and nurse and dermatologist supervision (aim and content of the CHT, see supplementary file 1). Previous studies have reported that CHT has positive effects on outcomes such as; disease severity,^13^, ^14^, ^15^, ^16^ mental health,^17^ level of knowledge,^18^ self‐management^15^ and health‐related quality of life.^16^

### 3.3 Ethics

The study was approved by the Regional Committee for Medical Research Ethics for Southern Norway (ID 2016/1745) and conducted following the Helsinki declaration.

### 3.4 Measures

Socio‐demographics included age, gender, education, marital status, years with psoriasis and the number of other diseases.

The HLQ^10^ includes 44 items over nine independent scales. Each scale represents a different element of the overall HL construct. The opening five scales comprise items that ask the respondents to indicate their level of agreement (scoring 1–4), and the remaining scales^6^, ^7^, ^8^, ^9^ embody ranges of self‐reported capability (scoring 1–5), see Table 1. A lower score indicates a lower HL. The full HLQ offers nine individual scores based on an average of the items within each of the nine scales, with higher scores indicating higher HL.

**TABLE 1:** Health Literacy Questionnaire (HLQ) scales with the number of items and range of response categories (Norwegian version)

Scales	Number of items	Response scale
1. Feeling understood and supported by health‐care providers	4	1 = Strongly disagree; 2 = disagree; 3 = agree; 4 = strongly agree
2. Having sufficient information to manage my health	4	
3. Actively managing my health	5	
4. Social support for health	5	
5. Appraisal of health information	5	
6. Ability to actively engage with health‐care providers	5	1 = Cannot do; 2 = very difficult; 3 = quite difficult; 4 = quite easy; 5 = very easy
7. Navigating the health‐care system	6	
8. Ability to find good health information	5	
9. Understanding health information well enough to know what to do	5	

*The Self‐Administrated Psoriasis Area and Severity Index (SAPASI)* ^19^ measures disease severity, a structured instrument that allows subjects to assess accurately the severity of their psoriasis (score 0–72, where a higher score indicates more severe disease).

An adapted and simplified version of *the Self‐Administered Comorbidity Questionnaire (SCQ‐18)* measured medical comorbidity, where higher scores indicate a more severe comorbidity profile.^20^

Two scales (‘skill and technique acquisition’ and ‘self‐monitoring and insight’) from the *Health Education Impact Questionnaire (HeiQ)* measured self‐management.^21^ The scale scores range between 1 and 4. A higher score indicates better self‐management.

*The General Self‐efficacy (GSES) scale* measured self‐efficacy.^22^ The scale has 10 items with a response range from 1 (not at all true) to 4 (exactly true), and a higher score means higher self‐efficacy.

*The Psoriasis Knowledge Questionnaire (PKQ)* ^18^ assesses psoriasis knowledge based on 44 psoriasis statements. The total calculated score range is 0–44, where higher scores indicate higher levels of knowledge.

*Dermatology Life Quality Index (DLQI)* measured quality of life on a scale from 0 to 30.^23^ Higher scores specify larger impairment of a patient's quality of life.

*The Brief Illness Perception Questionnaire (BIPQ)* measures cognitive and emotional representations of illness.^24^ It is calculated as a single‐item scale approach to assessing perceptions on a scale from 0 to 10, where higher scores indicate stronger perceptions along that dimension.

### 3.5 Statistical analysis

Descriptive statistics report the characteristics of the study population. The expectation‐maximization (EM) algorithm was used to impute the missing HLQ item scores as previously employed by Beauchamp et al.^9^ For all HLQ scales, assumptions of normal distribution were met.

Using Ward's method, the CA was performed in Stata version 16 to identify and group participants with similar profiles of HL scores across the nine HLQ domains.^25^ Ward's Hierarchical Clustering Method measures cluster adequacy by evaluating distances between cluster centroids (a measure of cohesion) and different distances produce different cluster solutions.^26^ The clusters are presented as means (standard deviation [SD]) for each domain score in each cluster and accompanied by information about socio‐demographic distributions across the clusters. The method for choosing the number of clusters is guided by seeking to minimize the remaining variance within each scale within each cluster, as presented in earlier HLQ research.^27^ For example, if SD is greater than 0.6 for one or more of the scales, it may indicate that there is still significant subgroups within the cluster and ensuring that clusters represent different patterns of needs and strengths across the nine HLQ domains.

Following the CA, a regression analysis was performed investigating each clusters’ socio‐demographic profile and significant associations. The variables entered into the equations as independent variables were based on the arguments of factors associated with psoriasis or other chronic conditions and HL from preceding research (i.e., introduction). The choice of using the two‐step multiple regression analyses was done to see if variables were separately associated with socio‐demographic or with clinical variables.

The following two steps were performed with regard to entering independent variables into the regression analysis:

Step 1: Age, gender and high education as independent variables

Step 2: Step 1 + heiQ domains (self‐management), psoriasis knowledge (PKQ), number of diseases, SAPASI (psoriasis severity), self‐efficacy and quality of life (DLQI) as independent variables.

## 4 FINDINGS

### 4.1 Socio‐demographic and clinical characteristics

The participants had a mean age of 53.2 (SD 12.3) years, 47.5% were female, and they had a mean duration of psoriasis of 28 (SD 14.6) years ranging from 1 to 77 years (Table 2).

**TABLE 2:** The participants' demographic and clinical characteristics (*N* = 792) and Cronbach alpha values to assess internal consistency

		*N* (%)/mean (SD)/median (range)				
Female sex		376 (47.5%)				
Age (years)		53.2 (12.4)			
		(Range 18–83)			
**Marital status**						
Married/cohabiting		531 (67%)				
Unmarried/single		123 (15.5%)				
Divorced/separated/widowed		130 (16.4%)				
Others		9 (1.1%)				
Higher education (%)		314 (39.7%)				
Duration of disease (years)		27.8 (14.6)				
Health condition (VAS scale 0–100),		60.11 (SD 19.6)				
Self‐assessed health status (1–5 = poor–excellent)		3.32 (SD 0.92)				
Current smoker **YES**		190 (24.1%)				
Number of CHT treatment		2 (1–39)				
Biological medicines **YES**		112 (14.2%)				
Joint pain **YES**		529 (66.8%)				
Joint pain and PSA affirmed by rheumatologist **YES**		368 (46.5%)				
BMI		28.60 (5.30)				
Number of comorbidities		4.4 (2.5)				

		N (%)/mean (SD)/median (range)		Cronbach's alpha		
SAPASI (0–72; higher score = more serious disease)		7.49 (4.87)		0.74		
DLQI (0–30; higher score more impairment)		9.7 (0–30)		0.90		
PKQ (0–44; higher score = more knowledge)		24.8 (7.3)		NA		
GSE (10–40; higher score = higher self‐ efficacy)		30.20 (4.39)		0.85		
HeiQ: Self‐monitoring and insight (score 1–4, high score = good)		3.14 (0.42)		0.76		
HeiQ: Skill and technique acquisition (score 1–4, high score = good)		2.79 (0.53)		0.82		
Sum‐score BIPQ (0–80: higher scores reflect a more negative perception of psoriasis)		42.9 (10.8)		0.73		

Abbreviations: BIPQ, Brief Illness Perception Questionnaire; BMI, body mass index; DLQI, The Dermatology Life Quality Index; GSE, General Self‐efficacy Scale; PSA, psoriasis arthritis; SAPASI, Self‐Administrated Psoriasis and Severity Index; PKQ, Psoriasis Knowledge Questionnaire.

### 4.2 Clusters of health literacy with socio‐demographic profiles and significant associations

**FIGURE 1:**
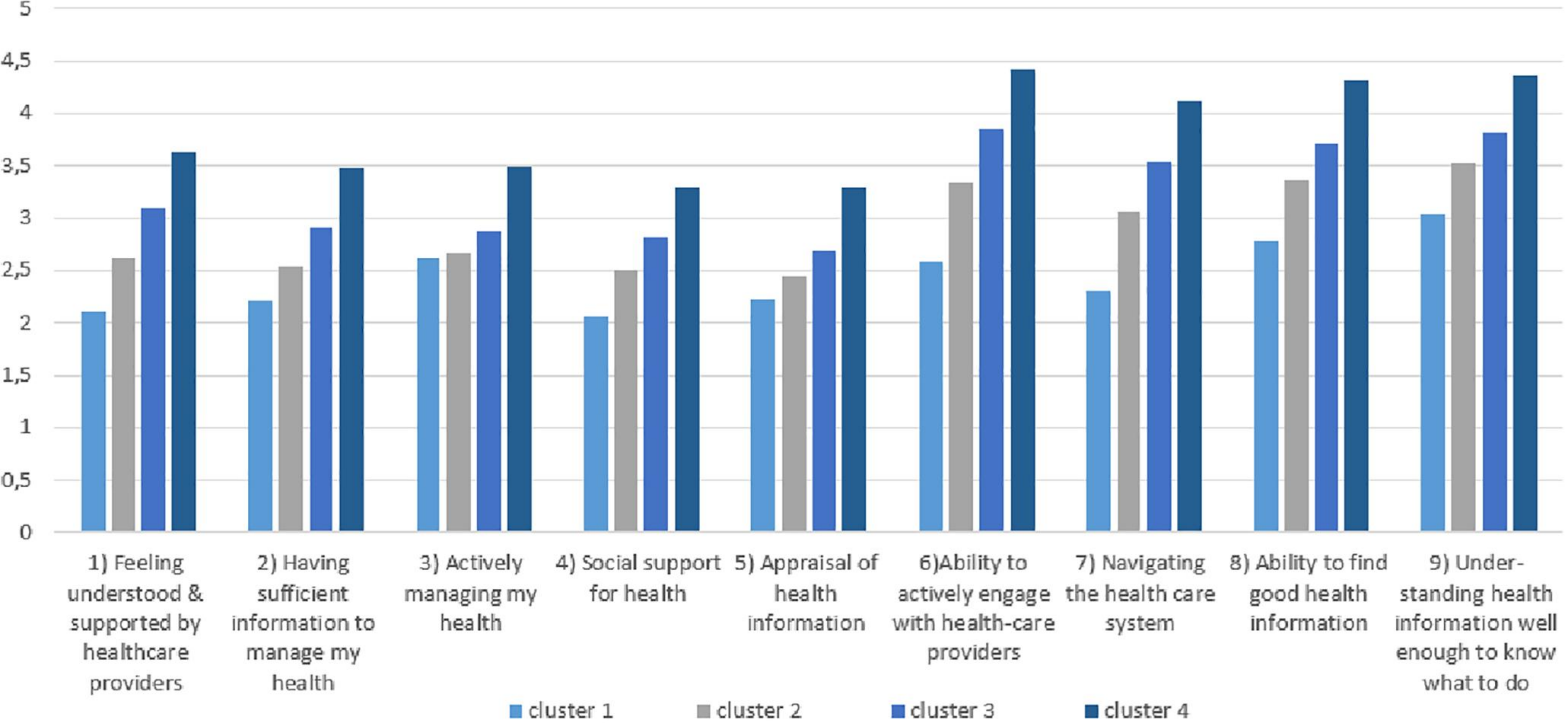
The clusters mean Health Literacy Questionnaire (HLQ) domain scores (about here)

In this sample, four clusters were chosen as the optimal cluster solution, based on cluster size and HL pattern diversity.^27^ These profiles ranged from people with lower HL who may require ongoing support to manage their health, through to people with higher HL who were more self‐confident users of health information and services. See Table 2 for more information on socio‐demographic characteristics and descriptive statistics. Table 3 presents mean HLQ scale scores of the total population with Cronbach alpha values. Each cluster presented a unique HLQ subscale pattern, also shown in Table 4 and Figure 1.

**TABLE 3:** Socio‐demographic characteristics and descriptive statistics related to the different clusters

	Cluster 1	Cluster 2	Cluster 3	Cluster 4
	Mean (SD) (*N* = 211) 26.6%	Mean (SD) (*N* = 258) 32.6%	Mean (SD) (*N* = 262) 33.1%	Mean (SD) (*N* = 61) 7.7%
Age (years)	53.46 (12.14)	52.80 (11.75)	53.71 (12.51)	51.59 (13.76)
Sex (% women)	48.34	46.12	46.95	52.46
Higher education (%)	31.90	38.76	42.75	57.38
Duration of disease (years)	26.96 (13.94)	27.82 (14.16)	28.17 (15.22)	29.45 (15.95)
SAPASI (0–72; higher score = more serious disease)	8.54 (5.22)	7.28 (4.52)	6.85 (4.56)	7.32 (5.77)
PKQ (0–44; higher score = more knowledge)	22.03 (6.72)	24.63 (7.09)	26.19 (6.44)	29.01 (6.47)
BIPQ (0–80: higher scores reflect a more negative perception of psoriasis)	48.22 (9.71)	43.79 (9.86)	39.89 (9.22)	33.47 (13.06)
Number of comorbidities (higher score = more comorbidity)	4.78 (2.63)	4.49 (2.48)	4.10 (2.43)	3.81 (2.14)
Quality of life (DLQI) (0‐30 higher score = more impairment)	12.25 (6.78)	9.61 (6.83)	8.36 (6.51)	6.76 (7.36)
HeiQ: Self‐monitoring and insight (score 1–4, high score = good)	2.97 (0.40)	3.04 (0.37)	3.25 (0.36)	3.64 (0.36)
HeiQ: Skill and technique acquisition (score 1–4, high score = good)	2.48 (0.53)	2.69 (0.46)	2.99 (0.35)	3.48 (0.52)

Abbreviations: BIPQ, Brief Illness Perception Questionnaire; DLQI: Dermatology Life Quality Index, HeiQ: Health Education Impact Questionnaire; SAPASI, Self‐assessed Psoriasis Area and Severity Index.

**TABLE 4:** Mean HLQ scale scores of the total population and clusters (*N* = 792)

	1) Feeling understood & supported by health‐care providers^1^, ^2^, ^3^, ^4^	2) Having sufficient information to manage my health^1^, ^2^, ^3^, ^4^	3) Actively managing my health^1^, ^2^, ^3^, ^4^	4) Social support for health^1^, ^2^, ^3^, ^4^	5) Appraisal of health information^1^, ^2^, ^3^, ^4^	6) Ability to actively engage with health‐care providers^1^, ^2^, ^3^, ^4^, ^5^	7) Navigating the health care system^1^, ^2^, ^3^, ^4^, ^5^	8) Ability to find good health information^1^, ^2^, ^3^, ^4^, ^5^	9) Under‐standing health information well enough to know what to do^1^, ^2^, ^3^, ^4^, ^5^
	Mean (Std)	Mean (Std)	Mean (Std)	Mean (Std)	Mean (Std)	Mean (Std)	Mean (Std)	Mean (Std)	Mean (Std)
Cluster 1 (*n* = 211)	2.11 (0.55)	2.22 (0.44)	2.61 (0.48)	2.06 (0.51)	2.23 (0.46)	2.58 (0.56)	2.30 (0.49)	2.79 (0.54)	3.04 (0.64)
Cluster 2 (*n* = 258)	2.62 (0.46)	2.53 (0.41)	2.66 (0.46)	2.51 (0.44)	2.45 (0.48)	3.34 (0.44)	3.06 (0.41)	3.36 (0.44)	3.52 (0.45)
Cluster 3 (*n* = 262)	3.09 (0.36)	2.91 (0.33)	2.88 (0.43)	2.82 (0.44)	2.69 (0.43)	3.85 (0.34)	3.55 (0.37)	3.72 (0.38)	2.66 (0.46)
Cluster 4 (*n* = 61)	3.64 (0.44)	3.48 (0.54)	3.49 (0.49)	3.30 (0.63)	3.30 (0.59)	4.42 (0.43)	4.12 (0.43)	4.31 (0.53)	4.37 (0.53)
Total sample (*N* = 792)	2.72 (0.65)	2.65 (0.54)	2.78 (0.51)	2.55 (0.59)	2.54 (0.54)	3.39 (0.73)	3.10 (0.71)	3.40 (0.64)	3.56 (0.62)

**Cluster 1** comprised 26.6% of the sample, and overall this group had lower HL. The HLQ domains from 1 to 5 have a mean score from 2.1 to 2.6 (possible scores from 1 to 4), and the domains from 6 to 9 have a mean score from 2.6 to 3.0 (possible scores from 1 to 5). The participants scored lowest in domain 1: Understood and supported by health providers (2.1, SD 0.55). They also had limited social support for health in domain 4 (2.1, SD 0.51) and were also not at all confident in their ability to navigate health services (scale 7) (2.30, SD 0.49). People in this cluster were not active information seekers (scale 8) and had little faith or confidence to build relationships with healthcare providers (HCP) (scales 1 and 6). In this cluster, the average age is 53 years, and 31% have a higher education. Further, the mean score in psoriasis knowledge (PKQ) was 22.0 (SD 6.7) (range 0–44), their mean score for illness perception (BIPQ) was 48.7 (SD 9.7) (range 0–80), and the mean number of comorbidities is 4.8.

The regression analysis (see Table 5) indicates that significant associations to this cluster in the second step was the skill and acquisition domain of self‐management (st.β −0.244), self‐efficacy (st.β −0.0081), psoriasis knowledge (st.β −0.0095) and quality of life measured by the DLQI (st.β.0075). This model explained 19.8% of the variance (adjusted *R*‐square), indicating that lower self‐management, lower self‐efficacy, lower psoriasis knowledge and lower quality of life, all were significantly associated with this cluster.

**Cluster 2** includes 32.6 % of the total sample. Here, the HLQ domains from 1 to 5 have a mean score from 2.5 to 2.7 (score 1–4), while the scores on domains 6– 9 (score 1–5), assessing HL tasks and skills, vary from 3.1 to 3.5. This cluster has the weakest ratings in domain 5, participants being unsure of where to find the reliable information (2.45, SD 0.48). They also reported problems in domain 7, being unclear about what health services were available (3.06, SD 0.41). The participants had an average age of 53.5 years, 46% were women, and 39% had a higher education. They had a mean score of 24.6 (SD 7.1) in psoriasis knowledge and a score of 43.8 (SD 9.9) in the BIPQ. The regression analysis shows that lower self‐monitoring and insight (st.β −0.160) related to self‐management is significantly associated with this cluster. Here, the variance explained by the model (adjusted R‐square) was 2%.

**Cluster 3** represents 33.1% of the sample. The HLQ domains’ 1–6 mean scores range from 2.7 to 3.1, and between 3.6 and 3.9 for the domains 6–9. The lowest scores are in HLQ domain 4, showing that the participants had limited social support for health (2.8, SD 0.44), and in domain 7, indicating that they were less confident about navigating the health care system (3.6, SD 0.37). Forty‐three per cent of the participants have a higher education. They generally score higher on psoriasis knowledge 26.2 (SD 6.4) and present better mean illness perception (39.9, SD 9.2) and fewer comorbidities (4.1). The regression analysis showed that higher age (st.β 0.03), higher scores on the skill and technique acquisition domain of self‐management (st.β: 0.013), and having more psoriasis knowledge (st.β: 0.007) were significantly associated to the cluster. Here the model explained 6.8% of the variance (see Table 5).

**TABLE 5:** Regression analysis with the clusters as the dependent variable (step 2)

Dependent variable	Cluster 1	Cluster 2	Cluster 3	Cluster 4
	St. beta (*p* value)	St. beta (*p* value)	St. beta (*p* value)	St. beta (*p* value)
Sex (men)	‐	‐	‐	‐
Higher education	‐	‐	‐	‐
Age (years) (higher value = higher age)	‐	‐	‐	‐
HeiQ: Self‐monitoring and insight	‐	−0.164 (0.003)		0.092 (0.002)
HeiQ: Skill and technique acquisition	−0.242 (<0.001)	‐	0.134 (0.004)	0.122 (< 0.001)
PKQ (higher score = more knowledge)	−0.0101 (< 0.001)	‐	0.0076 (0.008)	‐
Comorbidity (higher score = more comorbidities)	‐	‐	‐	‐
SAPASI (higher score = more severe disease)	‐	‐	‐	‐
Illness Perception (BIPQ)	‐	‐	‐	‐
Years with psoriasis	‐	‐	−0.003 (0.027)	‐
Quality of life (DLQI) (higher score = worse quality of life)	0.008 (0.007)	‐	‐	−0.0039 (0.024)
Self‐efficacy (GSM ) (higher score = better Se)	−0.0081 (0.035)	‐	‐	0.0055 (0.022)
Adjusted *R‐*square (%)	19.8%	2.0%	7.2%	17.5%

Abbreviations: DLQI, Dermatology Life Quality Index; HeiQ, Health Education Impact Questionnaire; PKQ, Psoriasis Knowledge Questionnaire; SAPASI, Self‐assessed Psoriasis Area and Severity Index.

**Cluster 4** is representing 7.7% of the sample. Overall, this small group had higher HL with a mean score on the HLQ domains 1–6 from 3.3 to 3.6. They were also confident users of the health system and health information (scales 6–9), with a mean score of 4.1–4.4. In this cluster, the lowest mean scores were related to the domain 4, where they reported limited social support for health (3.3, SD 0.63) and in domain 5, appraisal of health information (3.3, SD 0.59). The participants reported having good trust in health providers, being able to work collaboratively and having trust and confidence in building lasting relationships with HCP in HLQ domains 1 (3.6, SD 0.44) and 6 (4.4, SD 0.43). The participants' mean age was 52 years, 52.5% were women and 57% have a higher education. They have a mean score of 29.0 (SD 6.5) on psoriasis knowledge and 33.5 (SD 13.1) in illness perception. They had on average 3.8 comorbidities. The regression analysis (Table 5) shows that higher score on both self‐management domains; self‐monitoring and insight (st.β 0.092) and skill and technique acquisition (st.β 0.122, p), enhanced quality of life (DLQI) (st.β −0.0039) and better self‐efficacy (st.β 0.0045) all are associated with this cluster. The adjusted *R*‐square is 18.1%.

## 5 DISCUSSION

Our study generated a four‐cluster solution within the psoriasis sample, identifying clinically meaningful subgroups of patients. The results showed a diversity of HL profiles and revealed a pattern of low (Cluster 1) through to high (Cluster 4) HL based on a consistently mean of the nine HLQ scores. The main finding was that 26.6% of the psoriasis population belonged to the cluster with the lowest HLQ scores. This is in contrast to other HLQ studies within other chronic conditions (chronic kidney disease and cardiovascular disease), where the clusters with the lowest score are much smaller in percentage (respectively, 14 and 4.6%).^28^ ^,^ ^29^ The fact that as few as 7.7% of the psoriasis sample scores relatively high on most dimensions indicate that HL may be an essential area for increased focus also in general psoriasis care. These results may suggest that lower HL seems to be a more substantial problem in psoriasis, compared to other cohorts. The reason for this difference may be multifactorial; however, it may indicate that patients with psoriasis have less systematic healthcare support associated with their self‐management efforts, compared to other chronic conditions. There are several strengths related to this study, both the sample size and the response rate, together with the use of valid instruments indicate important methodological strengths, yielding safe generalized results.

Doing the CA provided a clearer picture of the particular HL subgroups and certified our ability to confirm their liability and further describe their specific challenges. For example, Cluster 1, representing 26.6 % of the sample, showed severe HL limitations that were associated with lower psoriasis knowledge, lower quality of life, self‐management and self‐efficacy. HL domains connected to cooperation with HCPs showed especially low scores. This means that these patients lack support from HCP as well as their social system.^10^ These results are in contrast to the findings in Clusters 3 and 4, where the cluster participants score relatively high on feeling understood and supported by HCP, and in their ability to actively engage with them, indicating a relatively satisfying relationship to healthcare personnel, a feeling of control in such relationships and of being empowered.^10^

Our analysis found that patients in the four subgroups also differed significantly concerning their associations to demographics and other relevant outcomes, and we found a strong predisposition towards poor health indicators in clusters with insufficient HL profiles. In general, there is a definite trend towards more adverse health outcomes in the clusters with many HL challenges. The cluster with the most inferior HLQ profile (Cluster 1) also showed significant negative associations to self‐management, psoriasis knowledge and quality of life. Also, in the study with chronic renal failure patients, the subgroup with the lowest HL profile scored significantly lower on quality of life compared to the mid‐level and high‐level clusters.^28^ In this cluster, we also found a lack of ability to engage with HCPs, to navigate the healthcare system, and with getting help from their social environment, indicating a need for increased initiative and support by the HCPs.^30^ However, the Cluster 1 group scored somewhat better in the actively managing my life domain. Maybe this is caused by a need to compensate for the lack of other support, being forced to take responsibility for their health and make their own health‐related decisions.^10^

The framework of HLQ^10^ and other studies^9^ ^,^ ^31^ has established that patients in each cluster should have at least some strengths, but also report limitations on other HL dimensions. However, in the psoriasis sample, the HL patterns appeared different, with the subgroups generally showing matching levels of HL in all nine dimensions. This somewhat different distribution pattern also seems to be the case in another Norwegian study within kidney disease.^28^ Clusters with above‐average health indicators (Clusters 3 and 4) exhibited relatively high mean scores in all nine HLQ scales. However, despite having the most advantageous HL profile, especially regarding active engagement with and feel supported by HCPs (HLQ scale 6 and 1), persons in Cluster 3 more often had poor health indicators than persons in Cluster 4. These findings are somewhat similar to the previously mentioned Danish study.^8^

A recent systematic review on the perspectives of HCP and patients on HL^32^ showed discouraging results. There are significant gaps in HL knowledge among HCP and patients and HCP's lack of awareness of HL definitions, as well as an understanding of the concept. Given the scarce focus on HL in psoriasis research, there may be an even more significant need within psoriasis care to educate the HCPs about HL on how to deliver effective health information to the patients. Furthermore, a possible barrier may also be negative attitudes shown by patients towards HL and HL screening that has been described in studies within different patient contexts.^33^ ^,^ ^34^ However, these studies have used measures primarily focusing on functional HL and peoples’ numeracy and reading‐related skills. Thus they are not considered comprehensive measures of the skills needed by individuals in the healthcare environment.^35^ In contrast, the HLQ is well developed and measures HL in a broad, subjective and generic matter.^10^ Much of the recent research in the HL field is at the group and population levels, but one study has demonstrated that the HLQ also has measurement veracity at the patient and clinician levels.^36^ After the patients completed the HLQ, the clinicians of each patient completed the questionnaire about their patient. As far as we know, such research has not been conducted within dermatology. However, future research could indicate important implications for the quality of care. For example, clinicians can use the HLQ to detect and discuss differences between their own perspectives about a patient's HL and the patient's perspective and identify patients who may benefit from tailored education or self‐management support.

There seems to be limited research on HL in other chronic, pruritic dermatoses, such as atopic eczema, nodular prurigo or lichen planus. One Korean study^37^ found that middle‐school children with atopic dermatitis had significantly lower e‐HL than those without the disease. A small study exploring HL in patients with epidermolysis bullosa^38^ found that 57.6% had inadequate HL in reading skills. There has been some research related to HL in education materials,^39^ but otherwise, there seems to be a novel research field to explore HL and important associations within chronic dermatological diseases.

This study has some limitations. We do not know whether the population participating in CHT is comparable to the Norwegian psoriasis cohort. The non‐responders of our study may mostly be CHT participants not responding positively to climate therapy. Due to the lack of ethical consent for non‐responders, we did not obtain data about this group. It is possible that we overestimate the level of HL in our sample due to the self‐report nature of the data collection, as people with very low HL may not participate in such a survey. Even if the survey does include satisfactory variations in demographic and clinical characteristics (Table 2), the participants are to a great deal middle‐aged, even if their ages range from 21 to ‐83. In addition, the cross‐sectional design makes any causal conclusions impossible.

We did not find statistically significant associations between any of the clusters and gender, education, comorbidity, SAPASI or illness perception. It is well known that psoriasis and depression amplify each other,^40^ and studies in other chronic conditions have shown that depression negatively correlates with HL.^41^ A limitation of this study is our inability to check whether HL correlates with depression or anxiety in this psoriasis cohort. For example, a Slovakian study^42^ exploring whether depression and anxiety mediate HL's association with diet non‐adherence in dialysed patients found that patients in the low and moderate HL groups were more likely to report both anxiety and depression. Hence, increased levels of depression and anxiety in patients with limited HL may reduce their capacities to find, understand and act upon health information even more, leading to less effective self‐management. Further studies seem needed to determine the connection between anxiety and depression levels and HL and examine the exact extent of HL needs on self‐management for patients with psoriasis.

## 6 CONCLUSIONS

The HL profiles have provided a thorough assessment of the context‐specific needs and HL challenges among people with psoriasis having participated in climate therapy. While some subgroups might have a similar ‘total score’, the actions for improving their outcomes would differ severely. Knowing these patterns can guide our development of tailored interventions. Particular attention should be given to vulnerable patients characterized by low self‐management skills and self‐efficacy, low psoriasis knowledge and impaired quality of life, that also score low on HL related to HCP and social support.

## CONFLICT OF INTEREST

The authors have no conflicts of interest to declare.

## Supporting information

Supplementary Material
