# Supplementary material for: Health literacy profiling in persons with psoriasis – A cluster analysis
Source: Skin Health Dis. 2021 Feb 18;1(2):e17. doi: 10.1002/ski2.17 (PMC9060070; doi:10.1002/ski2.17)
Supplement: Supplementary file 1 — Supplementary Material [file SKI2-1-e17-s001.pdf]

SKIN HEALTH AND DISEASE Open Access

Health literacy profiling in persons with psoriasis - a cluster analysis

Journal:	*Skin Health and Disease*
Manuscript ID	SHD-2020-0030.R1
Wiley - Manuscript type:	Original Article
Date Submitted by the Author:	05-Jan-2021
Complete List of Authors:	Larsen, Marie; Lovisenberg Diaconal University College, Mastersprogram ; University of Oslo, Institute of Health and Society, Department of Health Sciences<br>Hermansen , Åsmund ; Oslo Metropolitan University, Faculty of Social Sciences, Department of Social Work,<br>Borge, Christine; University of Oslo, Department of Health Sciences ; Lovisenberg Diakonale Hospital<br>Strumse, Yndis A ; Oslo Universitetssykehus, Section for climate therapy<br>Andersen , Marit; Oslo Universitetssykehus, Department of Transplantation Medicine ; University of Oslo , Institute of Health and Society, Depatment of Health Sciences<br>Wahl, Astrid; University of Oslo, Institute of Health and Society
Keywords:	Psoriasis, Autoinflammatory disorders, Quality of life, Psychodermatology
Abstract:	Objective: To explore HL profiles within a cohort of people with psoriasis. A cluster approach identifies groups of individuals that have similar health literacy (HL) profiles. The method unmasks sub-groups with particular HL strengths, or sub-groups with limitations, which require tailored health care services to improve.<br><br>Methods: A cross-sectional sample of 792 patients from the Norwegian Climate Helio Therapy Program in Gran Canaria participated. The Health Literacy Questionnaire assessed nine HL dimensions. Using Ward's Hierarchical Clustering Method (Stata version 16) we looked for subgroups of patients across the dimensions. We also explored whether these clusters had specific demographic features and associations to outcomes such as psoriasis knowledge, quality of life, and self-management capacity.<br><br>Result: The analysis revealed four unique clusters identifying clinically meaningful subgroups. Two groups stood out as especially interesting. One cluster representing 26.6 % of the sample presented severe HL limitations associated with lower psoriasis knowledge, quality of life, self-management, and self-efficacy. HL domains connected to cooperation with health care professionals showed deficient scores. The other cluster included a smaller percentage (7.7%) with high HL compared to the total sample. This cluster was associated with higher self-management, quality of life, and better self-efficacy.

Conclusion: The cluster analysis revealed substantial differences in HL profiles within the sample. These results support the importance of a holistic understanding of the HL needs and the vulnerabilities within a psoriasis cohort. Implementing one size fits all approaches, may not be sufficient in psoriasis context to target HL.

SCHOLARONE™
Manuscripts

Health literacy profiling in persons with psoriasis – a cluster analysis

M.H. Larsen,[1,2] Å. Hermansen,[3] C.R. Borge,[1,4] Y.S. Strumse,[5] M.H. Andersen[1,6] and A.K. Wahl[1,6]

[1] Lovisenberg Diaconal University College, Oslo, Norway

[2] Department of Interdisciplinary Health Sciences, Institute of Health and Society, University of Oslo, Oslo, Norway

[3] Faculty of Social Sciences, Oslo Metropolitan University, Oslo, Norway

[4] Lovisenberg Diaconal Hospital, Oslo, Norway

[5] Section for Climate Therapy, Oslo University Hospital, Oslo, Norway

Correspondence: Marie Hamilton Larsen

E-mail: m.h.larsen@medisin.uio.no / marie.h.larsen@ldh.no

ORCID: 0000-0001-9113-1062

Funding: None

Conflicts of interest: Authors have no conflicts of interest to declare.

For Review Only

What is already known about the topic?

- Health literacy (HL) is an essential factor for ensuring effective self-management of chronic conditions such as psoriasis.
- People with psoriasis have lower health literacy scores compared to other chronic conditions.
- Holistic care of people with psoriasis requires knowledge about health literacy, self-management support, and management of comorbidities and associated risk factors.

What does this study add?

For Review Only

- There are considerable differences in health literacy profiles within a psoriasis sample; having a low health literacy profile is associated with lower psoriasis knowledge, quality of life, self-efficacy, and self-management.
- People in low scoring clusters are not active information seekers, have low social support, and have limited faith in building relationships with health care providers.
- Our findings suggest that knowing health literacy profiles can guide the development of tailored health literacy interventions, securing high utility and uptake in the psoriasis context.

Abstract

Objective: To explore HL profiles within a cohort of people with psoriasis. A cluster approach identifies groups of individuals that have similar health literacy (HL) profiles. The method unmasks sub-groups with particular HL strengths, or sub-groups with limitations, which require tailored health care services to improve.

Methods: A cross-sectional sample of 792 patients from the Norwegian Climate Helio Therapy Program in Gran Canaria participated. The Health Literacy Questionnaire assessed nine HL dimensions. Using Ward's Hierarchical Clustering Method (Stata version 16) we looked for subgroups of patients across the dimensions. We also explored whether these clusters had specific demographic features and associations to outcomes such as psoriasis knowledge, quality of life, and self-management capacity.

Result: The analysis revealed four unique clusters identifying clinically meaningful subgroups. Two groups stood out as especially interesting. One cluster representing 26.6 % of the sample presented severe HL limitations associated with lower psoriasis knowledge, quality of life, self-management, and self-efficacy. HL domains connected to cooperation with health care professionals showed deficient scores. The other cluster included a smaller percentage (7.7%) with high HL compared to the total sample. This cluster was associated with higher self-management, quality of life, and better self-efficacy.

Conclusion: The cluster analysis revealed substantial differences in HL profiles within the sample. These results support the importance of a holistic understanding of the HL needs and the vulnerabilities within a psoriasis cohort. Implementing one size fits all approaches, may not be sufficient in psoriasis context to target HL.

For Review Only

Introduction

Psoriasis is a chronic inflammatory skin disease, and in later years, knowledge about pathogenesis and effective pharmacological treatment options have advanced significantly. Still, several critical knowledge gaps remain, and many patients lack an efficient treatment regime [1, 2]. One such knowledge gap is related to profiling the ability of patients to make use of health information related to psoriasis, named health literacy.

Health literacy (HL) refers to a person's ability to engage effectively with health information and services[3] and is a multidimensional concept covering functional, social, and critical dimensions [4]. Poor HL creates barriers to understand one's health, illness, and treatment fully. In the health literacy field, most of the research has been focusing on reading comprehension and numeracy skills, better known as "functional" HL[5]. However, measuring only functional HL overlooks the complexity of cultural and personal values, the importance of context, and the social resources and individual motivation that influence peoples` ability to understand and act upon information associated with their health [6]

Previous findings from this sample [7] showed that the participants with psoriasis generally scored low on most of the HL domains, also compared to people with other chronic conditions [8-10]. However, these results provided only information of the whole sample, and the linear regression models gave no indications to whether there were significant HL differences within the psoriasis cohort. To be able to examine the Health Literacy Questionnaire (HLQ) data[10] and reveal possible subgroups of participants, to use latent profiles or cluster analysis (CA) is recommended [11]. Conceptually, CA aims to identify cluster solutions that are relatively homogeneous within each group, leading to clusters that show high intra-class similarity, while maximizing heterogeneity between the groups, leading to low inter-class similarity across the clusters[12].

Hence, this study aimed to provide detailed profiles of HL strengths and weaknesses in the psoriasis cohort. The following research question is asked:

What types of HL profiles can be identified by investigating HLQ clusters and their characteristics in a sample of patients with psoriasis?

Methods

For Review Only

Patients and methods

A total of 792 participants > 18 years (65% response rate) provided sufficient data to be included in the cluster analysis. They had previously (once or several times from 2011 to 2017) participated in the Norwegian Climate Heliotherapy (CHT) program in Gran Canaria. They were by postal mail requested to partake, and a reminder letter was sent after six weeks. Data collection took place from March to August 2017.

The Climate Therapy Program (CHT)

Climate therapy/heliotherapy (CHT) comprises sunlight and saltwater treatment to relieve symptoms and is one of the therapeutic options available to Norwegian patients with moderate to severe psoriasis. CHT is provided in the Canary Islands (located in the Atlantic Ocean at 28°N, 16°W) and includes three weeks of individualized sun exposure in increasing doses as the primary treatment. Additionally, the program emphasizes daily physical training, tailored education, group discussions, individual consultations, and nurse and dermatologist supervision (Aim and content of the CHT see Supplementary file1). Previous studies have reported that CHT has positive effects on outcomes such as; disease severity [13, 14, 15, 16], mental health [17], level of knowledge [18], self-management [15] and health-related quality of life [16].

Ethics

The study was approved by the Regional Committee for Medical Research Ethics for Southern Norway (ID 2016/1745) and conducted following the Helsinki declaration.

Measures

Socio-demographics included age, gender, education, marital status, years with psoriasis, and the number of other diseases.

The Health Literacy Questionnaire (HLQ) [10] includes 44 items over nine independent scales. Each scale represents a different element of the overall HL construct. The opening five scales comprise items that ask the respondents to indicate their level of agreement (scoring 1 to 4), and the remaining scales (6–9) embody ranges of self-reported capability (scoring 1to 5). A lower score indicates a lower HL. The full HLQ offers nine individual scores based on an average of the items within each of the nine scales, with higher scores indicating higher health literacy.

Table 1: HLQ scales with the number of items and range of response categories (Norwegian version) (about here)

For Review Only

The Self-Administrated Psoriasis Area and Severity Index (SAPASI) [19] measures disease severity, a structured instrument that allows subjects to assess accurately the severity of their psoriasis (score 0-72, where a higher score indicates more severe disease).

An adapted and simplified version of *the Self-Administered Comorbidity Questionnaire (SCQ-18)* measured medical comorbidity, where higher scores indicate a more severe comorbidity profile[20].

Two scales ("Skill and technique acquisition" and "Self-Monitoring and insight") from the *Health Education Impact Questionnaire (HeiQ)* measured self-management [21]. The scale scores range between one and four. A higher score indicates better self-management.

The General Self-efficacy (GSES) scale measured self-efficacy[22] The scale has ten items with a response range from 1 (not at all true) to 4 (exactly true), and a higher score means higher self-efficacy.

The Psoriasis Knowledge Questionnaire (PKQ)[18] assesses psoriasis knowledge based on 44 psoriasis statements. The total calculated score range is 0–44, where higher scores indicate higher levels of knowledge.

Dermatology Life Quality Index (DLQI) measured quality of life on a scale from 0 to 30[23]. Higher scores specify larger impairment of a patient's quality of life.

The Brief Illness Perception Questionnaire (BIPQ) measures cognitive and emotional representations of illness[24]. It is calculated as a single-item scale approach to assessing perceptions on a scale from 0 to 10, where higher scores indicate stronger perceptions along that dimension.

For Review Only

Statistical analysis

Descriptive statistics report the characteristics of the study population. The expectation-maximization (EM) algorithm was used to impute missing HLQ item scores as previously employed by Beauchamp et al. [9]. For all HLQ scales, assumptions of normal distribution were met.

Using Ward's method, the cluster analysis was performed in Stata version 16 to identify and group participants with similar profiles of HL scores across the nine HLQ domains [25]. Ward's

Hierarchical Clustering Method measures cluster adequacy by evaluating distances between cluster centroids (a measure of cohesion) and different distances produce different cluster solutions [26]. The clusters are presented as means (SD) for each domain score in each cluster and accompanied by information about socio-demographic distributions across the clusters. The method for choosing the number of clusters is guided by seeking to minimize the remaining variance within each scale within each cluster, as presented in earlier HLQ research[27]. For example, if standard deviations (SD) are greater than 0.6 for one or more of the scales, it may indicate that there is still significant subgroups within the cluster and ensuring that clusters represent different patterns of needs and strengths across the nine HLQ domains.

Following the cluster analysis, a regression analysis was performed investigating each clusters` socio-demographic profile and significant associations. The variables entered into the equations as independent variables were based on the arguments of factors associated with psoriasis or other chronic conditions and health literacy from preceding research (i.e. introduction). The choice of using the two-step multiple regression analyses were done to see if variables were separately associated by socio-demographic or with clinical variables.

The following two steps were performed with regard to entering independent variables into the regression analysis:

Step 1: Age, gender and high education as independent variables

Step 2: Step1 + heiQ domains (self-management), Psoriasis knowledge (PKQ), number of diseases, SAPASI (psoriasis severity), self-efficacy and quality of life (DLQI) as independent variables.

For Review Only

Findings

Socio-demographic and clinical characteristics:

The participants had a mean age of 53.2 (SD 12.3) years, 47.5% were female, and they had a mean duration of psoriasis of 28 (SD 14.6) years ranging from one to 77 years (Table 2).

Table 2. *Demographics and PROM scores for the whole sample (about here)*

Clusters of health literacy with socio-demographic profiles and significant associations

In this sample, four clusters were chosen as the optimal cluster solution, based on cluster size and HL pattern diversity [27]. These profiles ranged from people with lower HL who may

require ongoing support to manage their health, through to people with higher HL who were more self-confident users of health information and services. See Table 2 for more information on socio-demographic characteristics and descriptive statistics. Table 3 presents mean HLQ scale scores of the total population with Cronbach Alpha values. Each cluster presented a unique HLQ subscale pattern, also shown in Table 4 and Figure 1.

Table 3. *Mean HLQ scores for the population with Chronbach Alpha values (about here)*

Table 4. *Demographics and PROM scoring for the four clusters (about here)*

Cluster 1 comprised 26.6% of the sample, and overall this group had lower HL. The HLQ domains from 1-5 have a mean score from 2.1 to 2.6 (possible scores from 1-4), and the domains from 6-9 have a mean score from 2.6-3.0 (possible scores from 1-5). The participants scored lowest in domain1: Understood and supported by health providers (2.1 (SD .55)). They also had limited social support for health in domain 4 (2.1 (SD.51)) and were also not at all confident in their ability to navigate health services (scale7), (2.30 (SD .49)). People in this cluster were not active information seekers (scale 8) and had little faith or confidence to build relationships with health care providers (HCP) (scales 1 and 6). In this cluster, the average age is 53 years, and 31% have a higher education. Further, the mean score in psoriasis knowledge (PKQ) was 22.0 (SD 6.7) (range 0-44), their mean score for illness perception (BIPQ) was 48.7 (SD 9.7) (range 0-80), and the mean number of comorbidities is 4.8.

For Review Only

The regression analysis indicates that significant associations to this cluster in the second step, was the skill and acquisition domain of self-management (st.β -.244), self-efficacy (st.β -.0081), psoriasis knowledge (st.β -.0095) and quality of life measured by the DLQI (st.β.0075). This model explained 19.8% of the variance (adjusted R-square), indicating that lower self-management, lower self-efficacy, lower psoriasis knowledge and lower quality of life, all were significantly associated with this cluster.

Cluster 2 includes 32.6 % of the total sample. Here, the HLQ domains from 1-5 have a mean score from 2.5 to 2.7 (score 1-4), while the scores on domains 6 to 9 (score 1-5), assessing HL tasks and skills, vary from 3.1 to 3.5. This cluster has the weakest ratings in domain 5, participants being unsure of where to find reliable information, (2.45, SD.48). They also reported problems in domain 7, being unclear about what health services were available (3.06, SD .41). The participants had an average age of 53.5 years, 46% were women, and 39% had a

higher education. They had a mean score of 24.6 (SD 7.1) in psoriasis knowledge and a score of 43.8 (SD 9.9) in the BIPQ. The regression analysis shows that lower self- monitoring and insight (st.β -.160) related to self-management is significantly associated with this cluster. Here, the variance explained by the model (adjusted R-square) was 2%.

Cluster 3 represents 33.1% of the sample. The HLQ domains` 1-6 mean scores range from 2.7 to 3.1, and between 3.6 and 3.9 for the domains 6 to 9. The lowest scores are in HLQ domain 4, showing that the participants had limited social support for health (2.8 (SD .44), and in domain 7, indicating that they were less confident about navigating the health care system (3.6, (SD .37)). 43 % of the participants have a higher education. They generally score higher on psoriasis knowledge 26.2 (SD 6.4) and present better mean illness perception (39.9, (SD 9.2)) and fewer comorbidities (4.1).The regression analysis showed that higher age (st.β 0.03), higher scores on the skill and technique acquisition domain of self- management (st.β: .013) and having more psoriasis knowledge (st.β: .007) were significantly associated to the cluster. Here the model explained 6.8% of the variance.

Cluster 4 is representing 7.7% of the sample. Overall, this small group had higher HL with a mean score on the HLQ domains 1-6 from 3.3 to 3.6. They were also confident users of the health system and health information (scales 6-9), with a mean score of 4.1 to 4.4. In this cluster, the lowest mean scores were related to the domains; 4, where they reported limited social support for health (3.3, SD.63) and in domain 5, Appraisal of health information (3.3, SD.59). The participants reported having good trust in health providers, being able to work collaboratively and having trust and confidence in building lasting relationships with HCP in HLQ domains 1 (3.6 (SD .44)), and 6 (4.4, (SD. 43)). The participants' mean age was 52 years, 52.5% were women, and 57% have a higher education. They have a mean score of 29.0 (SD 6.5) on psoriasis knowledge and 33.5 (SD 13.1) in illness perception. They had on average 3.8 comorbidities. The regression analysis shows that higher score on both self-management domains; self-monitoring and insight (st.β .092) and skill and technique acquisition (st.β .122, p), enhanced quality of life (DLQI) (st.β -.0039) and better self-efficacy (st.β .0045) all are associated with this cluster. The adjusted R-square is 18.1%.

Figure 1 *The clusters mean HLO domain scores (about here)*

Table 5. *The regression analyses, representing the significant associations between each cluster (dependent variable), demographics and other relevant outcomes (about here)*

For Review Only

Discussion

Our study generated a 4-cluster solution within the psoriasis sample, identifying clinically meaningful subgroups of patients. The results showed a diversity of HL profiles and revealed a pattern of low (Cluster 1) through to high (Cluster 4) health literacy based on a consistently mean of the nine HLQ scores. The main finding was that 26.6% of the psoriasis population belonged to the cluster with the lowest HLQ scores. This is in contrast to other HLQ studies within other chronic conditions (chronic kidney disease and cardiovascular disease), where the clusters with the lowest score are much smaller in percentage (respectively 14 and 4.6%) [28, 29]. The fact that as few as 7.7% of the psoriasis sample score relatively high on most dimensions indicate that HL may be an essential area for increased focus also in general psoriasis care. These results may suggest that lower HL seems to be a more substantial problem in psoriasis, compared to other cohorts. The reason for this difference may be multifactorial; however, it may indicate that patients with psoriasis have less systematic health care support associated with their self- management efforts, compared to other chronic conditions. There are several strengths related to this study, both the sample size and the response rate, together with the use of valid instruments indicate important methodological strengths, yielding safe generalized results.

Doing the cluster analysis provided a clearer picture of the particular HL subgroups and certified our ability to confirm their liability and further describe their specific challenges. For example, cluster 1, representing 26.6 % of the sample, showed severe HL limitations that were associated with lower psoriasis knowledge, lower quality of life, self-management and self-efficacy. HL domains connected to cooperation with HCPs showed especially low scores. This means that these patients lack support from HCP as well as their social system[10]. These results are in contrast to the findings in cluster 3 and 4 where the cluster participants score relatively high on feeling understood and supported by HCP, and in their ability to actively engage with them, indicating a relatively satisfying relationship to health care personnel, a feeling of control in such relationships and of being empowered[10].

Our analysis found that patients in the four subgroups also differed significantly concerning their associations to demographics and other relevant outcomes, and we found a strong predisposition towards poor health indicators in clusters with insufficient HL profiles. In general, there is a definite trend towards more adverse health outcomes in the clusters with many health literacy challenges. The cluster with the most inferior HLQ profile (cluster 1)

For Review Only

also showed significant negative associations to self-management, psoriasis knowledge and quality of life. Also, in the study with chronic renal failure patients, the subgroup with the lowest HL profile scored significantly lower on quality of life compared to the mid-level and high-level clusters[28]. In this cluster, we also found a lack of ability to engage with HCPs, to navigate the health care system, and with getting help from their social environment, indicating a need for increased initiative and support by the HCPs [30]. However, the cluster 1 group, scored somewhat better in the actively managing my life domain. Maybe this is caused by a need to compensate for the lack of other support, being forced to take responsibility for their health and make their own health-related decisions [10].

The framework of HLQ[10] and other studies[9, 31] has established that patients in each cluster should have at least some strengths, but also report limitations on other HL dimensions. However, in the psoriasis sample, the HL patterns appeared different, with the subgroups generally showing matching levels of HL in all nine dimensions. This somewhat different distribution pattern also seems to be the case in another Norwegian study within kidney disease [28] Clusters with above-average health indicators (Cluster 3 and 4) exhibited relatively high mean scores in all nine HLQ scales. However, despite having the most advantageous health literacy profile, especially regarding active engagement with and feel supported by health-care providers (HLQ scale 6 and 1), persons in Cluster 3 more often had poor health indicators than persons in Cluster 4. These findings are somewhat similar to the previously mentioned Danish study[8].

A recent systematic review on the perspectives of HCP and patients on HL[32] showed discouraging results. There are significant gaps in HL knowledge among HCP and patients and HCP''s lack of awareness of HL definitions, as well as an understanding of the concept. Given the scarce focus on HL in psoriasis research, there may be an even more significant need within psoriasis care to educate the HCPs about HLon how to deliver effective health information to the patients. Furthermore, a possible barrier may also be negative attitudes shown by patients towards HL and HL screening that has been described in studies within different patient contexts[33,34]. However, these studies have used measures primarily focusing on functional HL and peoples` numeracy and reading-related skills. Thus they are not considered comprehensive measures of the skills needed by individuals in the health care environment[35]. In contrast, the HLQ is well developed and measures HL in a broad, subjective, and generic matter[10]. Much of the recent research in the HL field is at the group and population levels, but one study has demonstrated that the HLQ also has measurement

For Review Only

veracity at the patient and clinician level [36]. After the patients completed the HLQ, the clinicians of each patient completed the questionnaire about their patient. As far as we know, such research has not been conducted within dermatology. However, future research could indicate important implications for the quality of care. For example, clinicians can use the HLQ to detect and discuss differences between their own perspectives about a patient's HL and the patient's perspective and identify patients who may benefit from tailored education or self-management support.

There seems to be limited research on health literacy in other chronic, pruritic dermatoses, such as atopic eczema, nodular prurigo or lichen planus. One Korean study [37] found that middle school children with atopic dermatitis had significantly lower e-health literacy than those without the disease. A small study exploring HL in patients with epidermolysis bullosa 38 found that 57.6% had inadequate health literacy in reading skills. There has been some research related to HL in education materials [39], but otherwise, there seems to be a novel research field to explore health literacy and important associations within chronic dermatological diseases.

For Review Only

This study has some limitations. We do not know whether the population participating in CHT are comparable to the Norwegian psoriasis cohort. The non-responders of our study may mostly be CHT participants not responding positively to climate therapy. Due to lack of ethical consent for non-responders, we did not obtain data about this group.It is possible that we overestimate the level of HL in our sample due to the self-report nature of the data collection, as people with very low HL may not participate in such a survey. Even if the survey does include satisfactory variations in demographic and clinical characteristics (Table 2), the participants are to a great deal middle-aged, even if their ages range from 21-83. In addition, the cross-sectional design makes any causal conclusions impossible.

We did not find statistically significant associations between any of the clusters and gender, education, comorbidity, SAPASI, or illness perception. It is well known that psoriasis and depression amplify each other [40], and studies in other chronic conditions have shown that depression negatively correlates with health literacy [41]. A limitation of this study is our inability to check whether HL correlates with depression or anxiety in this psoriasis cohort.. For example, a Slovakian study [42] exploring whether depression and anxiety mediate HL's association with diet non-adherence in dialyzed patients found that patients in the low and moderate HL groups were more likely to report both anxiety and depression. Hence, increased

levels of depression and anxiety in patients with limited HL may reduce their capacities to find, understand, and act upon health information even more, leading to less effective self-management. Further studies seem needed to determine the connection between anxiety and depression levels and health literacy and examine the exact extent of HL needs on self-management for patients with psoriasis.

Conclusions

The health literacy profiles have provided a thorough assessment of the context-specific needs and HL challenges among people with psoriasis having participated in climate therapy. While some subgroups might have a similar "total score," the actions for improving their outcomes would differ severely. Knowing these patterns can guide our development of tailored interventions. Particular attention should be given to vulnerable patients characterized by low self-management skills and self-efficacy, low psoriasis knowledge and impaired quality of life, that also score low on HL related to HCP and social support.

Acknowledgements: We wish to thank the participants who kindly participated in this study and the staff at The Climate therapy section at Oslo University Hospital who contributed to the data collection.

For Review Only

References

1. Albaghdadi A. Current and Under Development Treatment Modalities of Psoriasis: A Review. Endocr Metab Immune Disord Drug Targets. 2017;17(3):189-99.
2. Strober BE, van der Walt JM, Armstrong AW, Bourcier M, Carvalho AVE, Chouela E, et al. Clinical Goals and Barriers to Effective Psoriasis Care. Dermatol Ther 2018;9(1):5-18.
3. Baker DW. The meaning and the measure of health literacy. J Gen Intern Med. 2006;21. doi:10.1111/j.1525-1497.2006.00540.x.
4. Nutbeam D. The evolving concept of health literacy. Soc Sci Medicine. 2008;67(12):2072-8. doi:https://doi.org/10.1016/j.socscimed.2008.09.050.
5. Visscher BB, Steunenberg B, Heijmans M, Hofstede JM, Devillé W, van der Heide I, et al. Evidence on the effectiveness of health literacy interventions in the EU: a systematic review. BMC Public Health. 2018;18(1):1414.
6. Jessup RL, Osborne RH, Beauchamp A, Bourne A, Buchbinder R. Health literacy of recently hospitalized patients: a cross-sectional survey using the Health Literacy Questionnaire (HLQ). BMC Health Serv Res. 2017;17(1):52.
7. Larsen MH, Strumse YAS, Borge CR, Osborne R, Andersen MH, Wahl AK. Health literacy – a new piece of the puzzle in psoriasis care?, Br. J. Dermatol.. 2018, 180 (6), 1506-16, DOI: https://doi.org/10.1111/bjd.17595

8. Aaby A, Friis K, Christensen B, Rowlands G, Maindal HT. Health literacy is associated with health behaviour and self-reported health: A large population-based study in individuals with cardiovascular disease. Eur J Prev Cardiol. 2017;24(17):1880-8.
9. Beauchamp A, Buchbinder R, Dodson S, Batterham RW, Elsworth GR, McPhee C. Distribution of health literacy strengths and weaknesses across socio-demographic groups: a cross-sectional survey using the Health Literacy Questionnaire (HLQ). BMC Public Health. 2015;15.
10. Osborne RH, Batterham R, Elsworth G, Hawkins M, Buchbinder R. The grounded psychometric development and initial validation of the Health Literacy Questionnaire (HLQ). BMC Public Health. 2013;13,658 https://doi.org/10.1186/1471-2458-13-658

11. Goeman D, Conway S, Norman R, Morley J, Weerasuriya R, Osborne RH, et al. Optimizing Health Literacy and Access of Service Provision to Community Dwelling Older People with Diabetes Receiving Home Nursing Support. J Diabetes Res. 2016;2016:2483263.
12. Clatworthy J, Buick D, Hankins M, Weinman J, Horne R. The use and reporting of cluster analysis in health psychology: a review. Br J Health Psychol. 2005;10:329-58.
13. Mork C, Wahl A. Improved quality of life among patients with psoriasis after supervised climate therapy at the Canary Islands. J. Am. Acad. Dermatol.. 2002;47(2):314-6.
14. Søyland E, Heier I, Rodríguez-Gallego C, Mollnes TE, Johansen FE, Holven KB, et al. Sun exposure induces rapid immunological changes in skin and peripheral blood in patients with psoriasis. The Br. J. Dermatol 2011;164(2):344-55.
15. Wahl AK, Langeland E, Larsen MH, Robinson HS, Osborne RH, Krogstad AL. Positive changes in self-management and disease severity following climate therapy in people with psoriasis. Acta dermato-venereologica. 2015;95(3):317-21.
16. Wahl AK, Mork C, Cooper BA, Padilla G. No long-term changes in psoriasis severity and quality of life following climate therapy. Journal of the American Academy of Dermatology. 2005;52(4):699-701
17 Langeland E, Robinson HS, Moum T, Larsen MH, Wahl AK. Mental health among people with psoriasis undergoing patient education in climate therapy. Scandinavian journal of psychology. 2013;54(6):508-14.
18. Wahl AK, Moum T, Robinson HS, Langeland E, Larsen MH, Krogstad AL. Psoriasis Patients''Knowledge about the Disease and Treatments. Dermatol Res Pract. 2013;2013:921737. https://doi.org/10.1155/2013/921737
19. Puzenat E, Bronsard V, Prey S, Gourraud PA, Aractingi S, Bagot M, et al. What are the best outcome measures for assessing plaque psoriasis severity? A systematic review of the literature. JEADV. 2010;24 Suppl 2:10-6.
20 Sangha O, Stucki G, Liang MH, Fossel AH, Katz JN. The Self-Administered Comorbidity Questionnaire: a new method to assess comorbidity for clinical and health services research. Arthritis Rheum. 2003;49(2):156-63. doi:10.1002/art.10993
21. Osborne RH, Elsworth GR, Whitfield K. The Health Education Impact Questionnaire (heiQ): an outcomes and evaluation measure for patient education and self-management interventions for people with chronic conditions. Patient Educ Couns. 2007;66(2):192-201.
22. Luszczynska A, Scholz U, Schwarzer R. The General Self-Efficacy Scale: Multicultural Validation Studies. J Psychol. 2005;139(5):439-57.
23. Finlay AY, Khan GK. Dermatology Life Quality Index (DLQI)--a simple practical measure for routine clinical use. Clin Exp Dermato. 1994;19(3):210-6.
24 Broadbent E, Petrie KJ, Main J, Weinman J. The brief illness perception questionnaire. J Psychosom Res. 2006;60(6):631-7.

For Review Only

25. Batterham RW, Hawkins M, Collins PA, Buchbinder R, Osborne RH. Health literacy: applying current concepts to improve health services and reduce health inequalities. Public Health. 2016;132:3-12.
26. Landau SCS, I Cluster Analysis: Overview. . In: Peterson PB, E ; McGaw, B, editor. International Encyclopedia of Education, 3rd edition Oxford, UK: Elsevier Ltd.; 2010. p. 72-83.
27. Batterham RW, Buchbinder R, Beauchamp A, Dodson S, Elsworth GR, Osborne RH. The OPtimising HEalth LIterAcy (Ophelia) process: study protocol for using health literacy profiling and community engagement to create and implement health reform. BMC Public Health. 2014;14:694.
28. Stømer UE, Gøransson LG, Wahl AK, Urstad KH. A cross-sectional study of health literacy in patients with chronic kidney disease: Associations with demographic and clinical variables. Nursing Open. 2019;0(0).
29. Aaby A, Beauchamp A, O''Hara J, Maindal HT. Large diversity in Danish health literacy profiles: perspectives for care of long-term illness and multimorbidity. Eur. J Public Health. 2019; 30(1)75-80.
30. Martin LR, Williams SL, Haskard KB, Dimatteo MR. The challenge of patient adherence. Ther Clin Risk Manag. 2005;1(3):189-99.
31. Rademakers J, Heijmans M. Beyond Reading and Understanding: Health Literacy as the Capacity to Act. Int. J. Environ. Res.. 2018;15(8):1676.
32. Rajah R, Ahmad Hassali MA, Jou LC, Murugiah MK. The perspective of health-care providers and patients on health literacy: a systematic review of the quantitative and qualitative studies. Perspect Public Health. 2017;138(2):122-32.
33. Wolf MS, Williams MV, Parker RM, Parikh NS, Nowlan AW, Baker DW. Patients''Shame and Attitudes Toward Discussing the Results of Literacy Screening. J. Health Commun. 2007;12(8):721-32.
34. Hahn EA, Garcia SF, Du H, Cella D. Patient attitudes and preferences regarding literacy screening in ambulatory cancer care clinics. Patient Relat Outcome Meas. 2010;1:19-27.
35. Berkman ND, Sheridan SL, Donahue KE. Low health literacy and health outcomes: an updated systematic review. Ann Intern Med. 2011;55.
36. Hawkins, M., Gill, S. D., Batterham, R., Elsworth, G. R., & Osborne, R. H. The Health Literacy Questionnaire (HLQ) at the patient-clinician interface: a qualitative study of what patients and clinicians mean by their HLQ scores. BMC Health Serv Res. 2017;17(1):309. https://doi.org/10.1186/s12913-017-2254-8
37. Park BK. Factors Influencing eHealth Literacy of Middle School Students in Korea: A Descriptive Cross-Sectional Study. Healthc Inform Res. 2019;25(3):221-9.
38. Parvizi MM, Lankarani KB, Handjani F, Ghahramani S, Parvizi Z, Rousta S. Health literacy in patients with epidermolysis bullosa in Iran. J Educ Health Promot. 2017;6:105.
39. Sahi FM, Masood A, Danawar NA, Mekaiel A, Malik BH. Association Between Psoriasis and Depression: A Traditional Review. Cureus. 2020;12(8):e9708. https://doi.org/10.7759/cureus.9708
40. Prabhu AV, Gupta R, Kim C, Kashkoush A, Hansberry DR, Agarwal N, et al. Patient Education Materials in Dermatology: Addressing the Health Literacy Needs of Patients. JAMA Dermatol. 2016;152(8):946-7.
41. Wang, B, Xia,L Yu, J, Feng,Y, Hong, J, Wang, W. The multiple mediating effects of health literacy and self-care confidence between depression and self-care behaviors in patients with heart failure, Heart & Lung, 2020; 49(6): 842-47 https://doi.org/10.1016/j.hrtlng.2020.09.011.

For Review Only

42. Skoumalova I, Geckova AM, Rosenberger J, Majernikova M, Kolarcik P, Klein D, et al. Does Depression and Anxiety Mediate the Relation between Limited Health Literacy and Diet Non-Adherence? Int J Environ

For Review Only

Table 1. Health Literacy Questionnaire (HLQ) scales with the number of items and range of response categories (Norwegian version)

Scales	Number of items	**Response scale**
1. Feeling understood and supported by health-care providers	4	1 = Strongly disagree; 2 = disagree; 3 = agree; 4 = strongly agree
2. Having sufficient information to manage my health	4	
3. Actively managing my health	5	
4. Social support for health	5	
5. Appraisal of health information	5	
6. Ability to actively engage with health-care providers	5	1 = Cannot do; 2 = very difficult; 3 = quite difficult; 4 = quite easy; 5 = very easy
7. Navigating the health-care system	6	
8. Ability to find good health information	5	
9. Understanding health information well enough to know what to do	5	

For Review Only

Table 2. The participants' demographic and clinical characteristics (N= 792) and Cronbach alpha values to assess internal consistency.

	N (%) / mean(SD) / Median (Range)	
Female sex	376 (47.5%)	
Age (years)	53.2(12.4) (Range 18-83)	
**Marital status:**		
Married/cohabiting	531 (67 %)	
Unmarried/ Single	123 (15.5 %)	
Divorced/separated/widowed	130 (16.4 %)	
Others	9 (1.1 %)	
Higher Education (%)	314 (39.7%)	
Duration of disease (years)	27.8 (14.6)	
Health condition (VAS scale 0 -100),	60.11 (SD19.6)	
Self-assessed health status (1–5 = poor - excellent)	3.32 (SD 0.92)	
Current smoker **YES**	190 (24.1%)	
Number of CHT treatment	2 (1-39)	
Biological medicines **YES**	112 (14.2%)	
Joint pain **YES**	529 (66.8%)	
Joint pain & PsA affirmed by rheumatologist **YES**	368 (46.5%)	
BMI	28.60 (5.30)	
Number of comorbidities	4.4 (2.5)	
	N (%) / mean(SD) / Median (Range)	Cronbach's alpha
SAPASI (0-72; higher score = more serious disease)	7.49 (4.87)	0.74
DLQI (0-30; higher score more impairment)	9.7 (0-30)	0.90
PKQ (0-44; higher score = more knowledge)	24.8 (7.3)	NA
GSE (10- 40; higher score = higher self- efficacy)	30.20 (4.39)	0.85
HeiQ: Self- Monitoring and insight (score 1–4, high score = good)	3.14 (.42)	0.76
HeiQ: Skill and technique acquisition (score 1–4, high score = good)	2.79 (.53)	0.82
Sum-score BIPQ (0- 80: higher scores reflect a more negative perception of psoriasis)	42.9 (10.8)	0.73

PsA= Psoriasis arthritis, Body Mass Index (BMI), Self-Administrated Psoriasis and Severity Index (SAPASI), The Dermatology Life Quality Index (DLQI), Psoriasis Knowledge Questionnaire (PKQ), General Self efficacy Scale (GSE) Brief Illness Perception Questionnaire (BIPQ).

For Review Only

Table 3. Socio-demographic characteristics and descriptive statistics related to the different clusters

	**Cluster 1** Mean (SD) (N= 211) 26.6%	**Cluster 2** Mean (SD) (N=258) 32.6%	**Cluster 3** Mean (SD) (N= 262) 33.1%	**Cluster 4** Mean (SD) (N= 61) 7.7%
**Age (years)**	53.46 (12.14)	52.80 (11.75)	53.71 (12.51)	51.59 (13.76)
**Sex (% women)**	48.34	46.12	46.95	52.46
**Higher education (%)**	31.90	38.76	42.75	57.38
**Duration of disease (years)**	26.96 (13.94)	27.82 (14.16)	28.17 (15.22)	29.45 (15.95)
**SAPASI** (0-72; Higher score = more serious disease)	8.54 (5.22)	7.28 (4.52)	6.85 (4.56)	7.32 (5.77)
**Psoriasis Knowledge (PKQ)** (0-44; higher score = more knowledge)	22.03 (6.72)	24.63 (7.09)	26.19 (6.44)	29.01 (6.47)
**Sum-score Brief Illness Perception Questionnaire (BIPQ)** (0- 80: higher scores reflect a more negative perception of psoriasis)	48.22 (9.71)	43.79 (9.86)	39.89 (9.22)	33.47 (13.06)
**Number of comorbidities** (higher score= more comorbidity)	4.78 (2.63)	4.49 (2.48)	4.10 (2.43)	3.81 (2.14)
**Quality of life (DLQI)** (0-30 Higher score = more impairment)	12.25 (6.78)	9.61 (6.83)	8.36 (6.51)	6.76 (7.36)
**HeiQ: Self- Monitoring and insight** (score 1–4, high score = good)	2.97 (.40)	3.04 (.37)	3.25 (.36)	3.64 (.36)
**HeiQ: Skill and technique acquisition** (score 1–4, high score = good)	2.48 (.53)	2.69 (.46)	2.99 (.35)	3.48 (.52)

SAPASI: Self-assessed Psoriasis Area and Severity Index; DLQI: Dermatology Life Quality Index, HeiQ: Health Education Impact Questionnaire

Only

Table 4. Mean HLQ scale scores of the total population and clusters (N=792)

	1) Feeling understood & supported by health-care providers (1-4)	2) Having sufficient information to manage my health (1-4)	3) Actively managing my health (1-4)	4) Social support for health (1-4)	5) Appraisal of health information (1-4)	6)Ability to actively engage with health-care providers (1-5)	7) Navigating the health care system (1-5)	8) Ability to find good health information (1-5)	9) Under-standing health information well enough to know what to do (1-5)
	**Mean (Std)**	**Mean (Std)**	**Mean (Std)**	**Mean (Std)**	**Mean (Std)**	**Mean (Std)**	**Mean (Std)**	**Mean (Std)**	**Mean (Std)**
**Cluster 1 (n=211)**	2.11 (.55)	2.22 (.44)	2.61 (.48)	2.06 (.51)	2.23 (.46)	2.58 (.56)	2.30 (.49)	2.79 (.54)	3.04 (.64)
**Cluster 2 (n=258)**	2.62 (.46)	2.53 (.41)	2.66 (.46)	2.51 (.44)	2.45 (.48)	3.34 (.44)	3.06 (.41)	3.36 (.44)	3.52 (.45)
**Cluster 3 (n=262)**	3.09 (.36)	2.91 (.33)	2.88 (.43)	2.82 (.44)	2.69 (.43)	3.85 (.34)	3.55 (.37)	3.72 (.38)	2.66 (.46)
**Cluster 4 (n=61)**	3.64 (.44)	3.48 (.54)	3.49 (.49)	3.30 (.63)	3.30 (.59)	4.42 (.43)	4.12 (.43)	4.31 (.53)	4.37 (.53)
**Total sample (N = 792)**	2.72 (.65)	2.65 (.54)	2.78 (.51)	2.55 (.59)	2.54 (.54)	3.39 (.73)	3.10 (.71)	3.40 (.64)	3.56 (.62)

Review Only

Table 5: Regression analysis with the clusters as the dependent variable (step 2)

Dependent variable	**Cluster 1**<br>**St. beta (p value)**	**Cluster 2**<br>**St. beta (p value)**	**Cluster 3**<br>**St. beta (p value)**	**Cluster 4**<br>**St. beta (p value)**
Sex (men)	-	-	-	-
Higher education	-	-	-	-
Age (years) (higher value= higher age)	-	-	-	-
HeiQ: **Self- Monitoring and insight**	-	-.164 **(0.003)**		.092 **(0.002)**
HeiQ: **Skill and technique acquisition**	-.242 **(<0.001)**	-	.134 **(0.004)**	.122 **(< 0.001)**
Psoriasis Knowledge (PKQ) (higher score= more knowledge)	-.0101 **(< 0.001)**	-	.0076 **(0.008)**	-
Comorbidity (higher score = more comorbidities)	-	-	-	-
SAPASI (higher score=more severe disease)	-	-	-	-
Illness Perception (BIPQ)	-	-	-	-
Years with psoriasis	-	-	-.003 **(0.027)**	-
Quality of life (DLQI) (higher score = worse quality of life)	.008 **(0.007)**	-	-	-.0039 **(0.024)**
Self-efficacy (GSM) (higher score= better Se)	-.0081 **(0.035)**	-	-	.0055 **(0.022)**
**Adjusted R Square (%)**	19.8%	2.0%	7.2%	17.5%

Psoriasis Knowledge Questionnaire; SAPASI: Self-assessed Psoriasis Area and Severity Index; DLQI: Dermatology Life Quality Index, HeiQ: Health Education Impact Questionnaire

Only

Supplementary file 1: Aim and content of the 3-week Climate Therapy Program in Gran Canaria

Aim:

- To reduce disease severity.
- Increase knowledge and insight about coping of the disease.
- Increase knowledge and insight about nutrition and physical activity.

Content:

- A combination of tailored sun treatment and salt water bathing
- Physical activity. Mandatory 30 minutes morning gym. Voluntary participation in other physical activities such as water gym, mountain trips and strength athletics.
- Multidisciplinary teaching in topics such as liniment, sun treatment, nutrition and physical activity.
- A total of 3-5 Group Meetings focusing on topics such as experiences in living with psoriasis, coping with stress, nutrition in daily life, sleep and rest and self-care.
- Self- management support with focus on body and soul and harmonious life style in a quiet atmosphere.

For Review Only

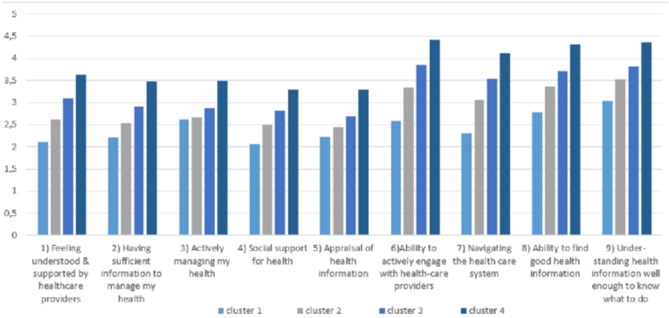

Figure 1: The clusters scoring on the nine HLQ domains

210x297mm (200 x 200 DPI)
